# Comorbidities Affect Risk of Nonvariceal Upper Gastrointestinal Bleeding

**DOI:** 10.1053/j.gastro.2013.02.040

**Published:** 2013-06

**Authors:** Colin John Crooks, Joe West, Timothy Richard Card

**Affiliations:** 1 Division of Epidemiology and Public Health, The University of Nottingham, Nottingham City Hospital, Nottingham, UK; 2 Nottingham Digestive Diseases Centre, National Institute for Health Research Biomedical Research Unit, Queen's Medical Centre, Nottingham University Hospitals National Health Service Trust, Nottingham, UK

**Keywords:** Etiology, Gastrointestinal Bleeding, Stomach, GIB, gastrointestinal bleeding, GPRD, General Practice Research Database, ITU, intensive therapy unit, NSAID, nonsteroidal anti-inflammatory drug, OR, odds ratio, PAF, population attributable fraction, PPI, proton pump inhibitors

## Abstract

**Background & Aims:**

The incidence of upper gastrointestinal bleeding (GIB) has not been reduced despite the decreasing incidence of peptic ulcers, strategies to eradicate *Helicobacter pylori* infection, and prophylaxis against ulceration from nonsteroidal anti-inflammatory drugs. Other factors might therefore be involved in the pathogenesis of GIB. Patients with GIB have increasing nongastrointestinal comorbidity, so we investigated whether comorbidity itself increased the risk of GIB.

**Methods:**

We conducted a matched case-control study using linked primary and secondary care data collected in England from April 1, 1997 through August 31, 2010. Patients older than 15 years with nonvariceal GIB (n = 16,355) were matched to 5 controls by age, sex, year, and practice (n = 81,636). All available risk factors for GIB were extracted and modeled using conditional logistic regression. Adjusted associations with nongastrointestinal comorbidity, defined using the Charlson Index, were then tested and sequential population attributable fractions calculated.

**Results:**

Comorbidity had a strong graded association with GIB; the adjusted odds ratio for a single comorbidity was 1.43 (95% confidence interval [CI]: 1.35–1.52) and for multiple or severe comorbidity was 2.26 (95% CI: 2.14%–2.38%). The additional population attributable fraction for comorbidity (19.8%; 95% CI: 18.4%–21.2%) was considerably larger than that for any other measured risk factor, including aspirin or nonsteroidal anti-inflammatory drug use (3.0% and 3.1%, respectively).

**Conclusions:**

Nongastrointestinal comorbidity is an independent risk factor for GIB, and contributes to a greater proportion of patients with bleeding in the population than other recognized risk factors. These findings could help in the assessment of potential causes of GIB, and also explain why the incidence of GIB remains high in an aging population.

See Covering the Cover synopsis on page 1327.

Helicobacter pylori infection, nonsteroidal anti-inflammatory medications (NSAIDs), and aspirin are believed to be the main causes of nonvariceal upper gastrointestinal bleeding,^1^ and with the discovery of proton pump inhibitors (PPIs) and *H pylori* eradication therapy, the burden of peptic ulcer disease has been decreasing.^2^ Despite this, upper gastrointestinal hemorrhage remains the most common acute severe medical admission for gastroenterology,^3,4^ and its incidence in population-based studies remains virtually unchanged.^5,6^ This suggests that other (previously unidentified) risk factors are contributing to its population burden.

Historically, nongastrointestinal comorbidity was believed to be associated with stress ulceration^7^ but, currently, the role of comorbidity in the etiology of gastrointestinal bleeding (GIB) is not recognized apart from in severe illness; for example, sicker cirrhotic patients are known to have an increased risk of variceal bleeding,^8^ and sicker patients in intensive therapy units (ITUs) have an increased risk of nonvariceal bleeding.^9^ However, as the proportion of bleed patients with comorbidity has increased during the last decade,^5^ we wondered if exposure to less severe but chronic comorbidity could itself be responsible for the persisting incidence of bleeding. Outside of ITU though, the effect of comorbidity has only been assessed as a confounder in studies that focused on the effect of medications on gastrointestinal bleeds.^10^ Although these studies do support a role for comorbidity, they do not allow us to understand whether it is an important independent contributor to the persisting burden of upper GIB.

We have therefore conducted a study aimed primarily at assessing whether comorbidity might have an important role in the etiology of upper GIB. To do this we have conducted a case-control study and formed a model fully corrected for known measured risk factors of upper GIB. We have then calculated the additional explanatory effect of adding comorbidity to our model to understand its effect on bleeding incidence in the general population.

## Methods

### Study Design

We conducted a matched case control study.

### Data

To provide the detailed longitudinal data and necessary power for this study, we have used the recently linked English Hospital Episodes Statistics data (secondary care data) and General Practice Research Database (GPRD) (primary care data). Because of the comprehensive English primary care system, the population registered to the GPRD is representative of the general English population.^11^ The data are subject to quality checks and a practice's data are only used when they are of high enough quality to be used in research, at these times the data are said to be “up to research standard.”^12^ The GPRD has been extensively validated for a wide range of diagnoses, with a mean positive predictive value of 89%.^13^ Ethical approval for this study was obtained from the Independent Scientific Advisory Committee for Medicines and Healthcare products Regulatory Agency database research. Fifty-one percent of English practices in GPRD have consented to record level linkage of their population to Hospital Episodes Statistics. This records all hospital admissions from the population registered to one of the linked primary care practices contributing to the GPRD. For this study, the linked dataset was available between April 1, 1997 and August 31, 2010.

### Case Definition

We have previously published the codes and methods used to define upper gastrointestinal bleeds in this study.^14^ In brief, we selected as exposed all patients with a first nonvariceal upper gastrointestinal bleed. A bleed was defined by a specific code for an upper gastrointestinal nonvariceal bleed in either primary or secondary care who had a supporting code in the linked dataset (defined as a likely symptom, cause, therapy, investigation, or outcome of upper gastrointestinal hemorrhage). Variceal bleeds or nonspecific gastrointestinal bleed codes with either a lower gastrointestinal diagnosis or procedure were excluded. Further exclusions were temporary patients (patients not registered permanently at a GPRD primary care practice, who might just be visiting the area of the practice briefly, and who are therefore not part of the GPRD's underlying population), children younger than 16 years old, cases with invalid date codes, or cases outside the up-to-research-standard observed time periods. Patients were required to be registered with the primary care practice for at least 3 months before an upper gastrointestinal bleed event to avoid including prevalent cases that might have been coded at the initial registration consultation. Only the first event for each patient was included. We have previously demonstrated that this selection strategy minimizes selection bias in studies of upper GIB in these data.^14^ A secondary analysis was then stratified by whether the defining bleed code or supporting code specifically referred to a peptic ulcer (Read codes J11 to J14 or *International Classification of Diseases*, 10^th^ Revision codes K25–K28). The Read codes had high positive predictive values (>95%) for peptic ulcers and upper gastrointestinal complications when validated in English primary care routine records.^15,16^

### Matched Controls

Each case was age (±5 years) and sex matched without replacement to 5 controls selected randomly who were alive at the time of the gastrointestinal bleed and registered to the same primary care practice. Controls were required to have been registered with the primary care practice for at least 3 months before the match date to be consistent with the definition for cases.

### Exposures

**Figure 1:**
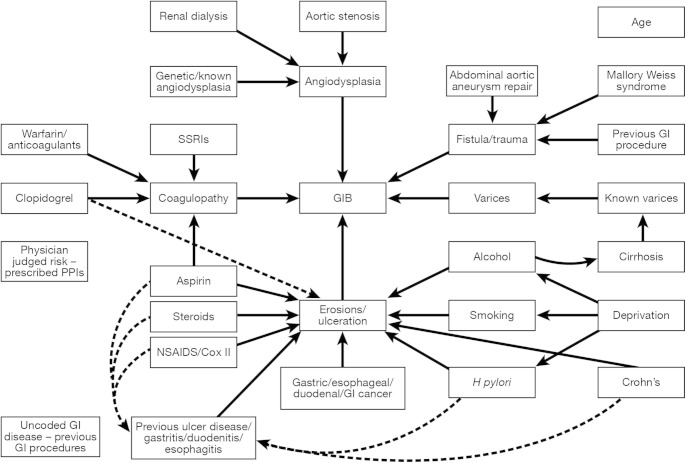
Risk factors for upper GIB. SSRI, selective serotonin reuptake inhibitor.

Potential final common causal pathways of an upper gastrointestinal bleed were defined a priori for erosions/ulceration, varices, angiodysplasia, fistula/trauma and coagulopathy, and code lists derived for diagnoses and medications that might be associated with each pathway based on published literature (Figure 1). Although variceal bleeds were excluded from the cases and controls, cirrhosis itself was included as a risk factor, as cirrhotic patients can have nonvariceal bleeds. Medication risk factors were included if there was a coded prescription within the year before the admission. Exposures coded within 2 months of the admission date were excluded to avoid identifying events and prescriptions related to the actual bleed event. PPIs were included as an indicator of physicians' judgement of the risk of upper gastrointestinal hemorrhage that was not captured by other measured risk factors. Alcohol consumption was classified as either nondrinker, alcohol mentioned, ex–alcohol dependency, alcohol excess, alcohol complications, and missing. Smoking was classified as never smoked, current smoker, ex-smoker, and missing. Cirrhosis was classified as uncomplicated, with varices, with ascites, or with encephalopathy or liver failure coded. All other exposures were binary variables.

### Comorbidity

Comorbidity was defined using the Charlson Index.^17^ This is a well-validated weighted comorbidity score derived from unselected hospital admissions that predicts 1-year mortality after hospital discharge. It has since been used in many contexts and has repeatedly measured the burden of comorbidity reliably. The original article demonstrated a graded increase in the risk in mortality associated with an increase in total score. The different comorbidities were assigned weights of 1, 2, 3, and 6, depending on their association with mortality. Where a graded effect was observed within a disease, for example, in diabetes or malignancy, these diseases were further stratified according to their severity. The conditions included in the original score (in order of weighting) were myocardial infarction, congestive heart failure, peripheral vascular disease, cerebrovascular disease, dementia, chronic pulmonary disease, connective tissue disease, peptic ulcer disease, mild liver disease, diabetes, hemiplegia, moderate or severe renal disease, diabetes with end organ damage, leukemia, lymphoma, moderate or severe liver disease, metastatic solid tumor, and acquired immunodeficiency syndrome. For our study, any codes already used to define risk factors of upper GIB in Figure 1 were excluded when calculating the index, ie, peptic ulcer and cirrhosis codes. For clarity in reporting in the tables, the index was summarized as no comorbidity (Charlson Index = 0), single comorbidity (Charlson Index = 1), and multiple or severe comorbidity (Charlson Index = 2).

### Analysis

#### Unadjusted analysis

Unadjusted odds ratios (ORs) were calculated for each exposure using conditional logistic regression to allow for the matched study design.

#### Multivariable analysis

Adjusted ORs for each exposure of interest were calculated with conditional logistic regression adjusting for all exposures in addition to age, PPI use, and previous gastrointestinal procedures. As calendar year, sex, and primary care practice were precisely matched on in the controls, it was not necessary to include them in the model. Comorbidity was added last, and its association with bleeding tested using a likelihood ratio test. The variance inflation factor (a measure of the increase in model variance due to correlation between variables) was calculated for each exposure of interest to assess the effect of correlation between variables. All exposures with a variance inflation factor >5 were excluded from the final conditional logistic regression model.^18^ The final model was then stratified into cases with a recording of peptic ulcer and those without.

### Sequential (or Extra) Population Attributable Fractions

Sequential (or extra) population attributable fractions (PAFs) were calculated for each exposure, using the prevalence among the cases and the respective coefficients from the conditional logistic regression model.^19^ Sequential PAFs differ from the standard adjusted PAFs that are usually presented. They are calculated by estimating the additional proportion of cases attributable to each exposure, after removing the proportion of cases already attributed to the combined effect of all other exposures in the model. The final model was then stratified into cases with a recording of peptic ulcer and those without. All analysis was performed using Stata software, version 12 (StataCorp LP, College Station, TX).

### Sensitivity Analyses

Previous studies of risk factor medications, such as NSAIDs,^20^ have been conducted in study populations that excluded patients with known risk factors for GIB. To allow comparisons with these, we re-estimated the crude ORs for each of the risk factor medications after excluding any cases and their controls with nonmedication bleed risk factors. To assess the effect of the choice of the exposure exclusion time window before the bleed event on the effect of NSAIDs, we also re-estimated a model that included NSAID use up to 30 days before the index date.

Two additional sensitivity analyses were performed to assess the effect of potential under-reporting. First the analysis was restricted to those older than 65 years old and who were eligible for free prescriptions, to assess the effect of potential under-reporting of nonprescribed NSAID use. Secondly, multiple imputation was used to re-estimate the association with comorbidity by imputing missing values for alcohol and smoking status. Alcohol and smoking were categorised as binary exposures of excess alcohol or current smoking to fit the logistic regression imputation model. All previously extracted exposures were used in the imputation model with addition of the socioeconomic status, and 20 sets of imputations were calculated. Socioeconomic status was measured by the Index of Multiple Deprivation quintiles obtained from linked Office of National Statistics data.

Finally, to assess the effect of using the aggregated and weighted Charlson Index, the model was re-estimated to assess the effect of the individual component comorbidities from the Charlson Index.

## Results

### Cases and Matching

There were 16,355 unique cases identified with a first nonvariceal bleed; 13,372 with specific code in Hospital Episodes Statistics, 10,938 with a specific code in GPRD, and 7955 with a specific code in both datasets. There were 16,304 (99.7%) cases matched to 5 controls each and only 8 cases (0.05%) were not matched to any controls. Median observed time before admission for cases was 7.4 years (interquartile range, 3.4–11.5) compared with 7.5 years (interquartile range, 3.5–11.5) for controls.

### Unadjusted Analysis

Table 1 shows the proportion of cases and controls with each exposure. As expected, aspirin and NSAIDs were the most frequently prescribed risk factor medications, and peptic ulcer and gastritis/duodenitis/esophagitis were the most frequent risk factor diagnoses. All a priori risk factors were associated with upper GIB. Peptic ulcers were coded as a diagnosis within the linked data in 4,823 patients (29% of cases). The exposures stratified by coding of peptic ulcer are shown in Supplementary Table 1.

### Multivariable Analysis and PAFs

There was strong evidence for an association between the nongastrointestinal Charlson Index and upper GIB after adjusting for all measured risk factors (single comorbidity adjusted OR = 1.43; 95% CI: 1.35–1.52; multiple or severe comorbidity adjusted OR = 2.26; 95% CI: 2.14–2.38; *P* < .001 likelihood ratio test). Table 2 shows the adjusted ORs from the final model for each exposure. We found the largest association with a bleed was with a previous Mallory-Weiss syndrome, which reflects the inherent risk of bleeding in recurrent vomitters. The variables for angiodysplasia and dialysis had the highest variance inflation factors, 1.48 and 2.35, respectively. As both of these were less than the a priori threshold of 5, all exposures were included in the final conditional logistic regression model.

Stratifying this model demonstrated similar associations with comorbidity, whether or not peptic ulcer coding was present, and slightly higher associations for a peptic ulcer with exposure to previous peptic ulcers, NSAID, or aspirin use (Table 3). Associations with other risk factors were higher in the nonpeptic ulcer cohort.

The proportion of cases attributable in the population to the combined effect of all available measured exposures was 48%, not including the effect of nongastrointestinal comorbidity. The additional proportion of cases attributable to nongastrointestinal comorbidity (or the sequential PAF) was 20%, and this was higher in magnitude than for any other measured exposure (Table 4). The next largest PAFs were 3% for aspirin and NSAID use.

The PAF for comorbidity associated with peptic ulcer bleeds was slightly lower than that for nonulcer bleeds (18% vs 21%), with a higher contribution from previous peptic ulcer bleeds and aspirin and NSAIDs (Table 5). In contrast, for nonulcer bleeds, the PAF was slightly increased for gastrointestinal cancer, alcohol, anticoagulants, and selective serotonin reuptake inhibitors.

### Sensitivity Analyses

The crude ORs were re-estimated for medications after excluding cases with nonmedication risk factors and these are shown in Supplementary Table 2. NSAID use was strongly associated with bleeding, with an OR of 1.67, and this increased to 2.80 with the exclusion of nonmedication risk factors. The corresponding adjusted ORs associated with NSAIDs were 1.59 with nonmedication risk factors included and 1.73 without. Altering the exposure exclusion window for NSAIDs to 30 days rather than 60 days before the bleed slightly increased the effect of NSAIDS, but had only a minimal effect on the other results, including comorbidity (see Supplementary Table 3).

Restricting the analysis to those older than 65 years old increased the proportion of cases attributable to the combined effect of all exposures from 48% to 63%, and reduced the additional proportion of cases attributable to nongastrointestinal comorbidity from 19.8% to 16.1%. Re-estimating the model using multiple imputation for missing alcohol and smoking status (modeled as binary exposures) slightly reduced the PAF associated with comorbidity from 22.9% to 22.4%, but when alcohol and smoking status were omitted from the model, the PAF was almost unaltered at 22.2%. Finally, the full model was re-estimated for each component of the Charlson Index (Table 6). The contribution of these individual comorbidities was minimal in comparison with their combined weighted effect in the Charlson Index in the main analysis.

## Discussion

This study has demonstrated that a combined measure of nongastrointestinal comorbidity is a significant independent predictor of upper GIB, even after accounting for all other recognized and measured risk factors. In addition, it explained a greater proportion of the burden of bleeding than any other risk factor in the population. The effect of this combined measure of nongastrointestinal comorbidity was far in excess of that which would be expected from its constituent diseases.

The association of comorbidities with upper GIB has been studied previously, but only in smaller secondary care surveys with comorbidity as a confounder and not as the primary exposure. We searched PubMed using variants of comorbidity, etiology, causality, risk factors, and gastrointestinal hemorrhage; however, no studies were identified that set out to address the question of our article. Studies were most frequently designed to measure the association of a single medication while adjusting for any confounding by comorbidity.^21,22^

Two studies assessed a larger range of medications in cross-sectional hospital-based surveys.^10,23^ First, Weil found that only 2 comorbidities, heart failure and diabetes, contributed to upper GIB with adjusted PAFs of 5% and 4%, respectively.^23^ However, the study was retrospective, and with <1000 cases limiting its power. In contrast to the “extra PAF” we calculated, the adjusted PAFs in their article calculated the effect of each exposure in a pseudo-population with no other risk factors present, potentially overestimating the effect in the general population, in which a case can be caused by many risk factors. The second comparable paper of Gallerani et al found an association with comorbidity and a similar 2-fold increase in risk in those exposed to NSAIDs to what we found in our peptic ulcer cohort.^10^ However, it was also a retrospective survey–based study potentially subject to recall bias, and had <1000 cases. Furthermore, the authors did not separate out gastrointestinal comorbidity from nongastrointestinal comorbidity and used hospital controls, therefore limiting comparisons with our population-based study.

Other studies assessed higher alcohol intake,^24^ *H pylori*,^25^ smoking,^26^ acute renal failure,^27^ and acute myocardial infarction^28^ and found associations with upper GIB. But these studies were in small selected hospitalised cohorts (n < 1000 bleeds) with limited assessments of individual comorbidity and no measure of their PAFs.

Our study has a number of important strengths when compared with these previous works because we set out specifically to assess the degree to which nongastrointestinal comorbidity predicts nonvariceal upper GIB after removing the effects of all the available known risk factors in a much larger general population. In addition, we used a method of defining cases and exposures that utilized information from both primary and secondary care, thereby maximizing the evidence supporting each case while not excluding severe events.^14^ Furthermore, due to the comprehensive coverage of the English primary care system, our study's results are likely to be generalizable to the whole English population and, we believe, further afield. The linked dataset used for our study remained representative of the GPRD overall, as whole practices rather than individual patients declined or consented to the linkage. Consequently, we were able to estimate the additional attributable fraction for comorbidity in the English population that was not already attributable to other risk factors.^19^

As our study was one of the first to assess the effect of the burden of comorbidity as a risk factor for upper GIB, no measure of comorbidity had been specifically validated for this purpose. We decided to use the Charlson Index because it is a well-validated score for measuring comorbidity in many different contexts. Other comorbidity scores that could be used, such as the Elixhauser Index or a simple counts of diagnoses, have been used and validated less frequently and in fewer contexts. In addition, some of the other scores also include other outcomes, such as financial cost, which are not necessarily a measure of the severity of disease. The Charlson Index was therefore selected as the most appropriate comorbidity score for our study.

We do need to consider alternative explanations for our observed association of comorbidity with upper GIB. A potential weakness of our study is the inevitably imperfect data on some recognized risk factors that might have caused us to underestimate their importance. The GPRD contains comprehensive recording of all available diagnoses and prescriptions. However, under-reporting is likely to have occurred for *H pylori* infection, NSAID use, alcohol, and smoking. In the case of *H pylori*, there was inevitable under-reporting because there was no population screening. However, if the under-reporting of *H pylori* infection was to explain our study's findings, it would have to be strongly associated with comorbidity, and the evidence for this is conflicting and underpowered.^29,30^ In studies of ischemic heart disease, for which there is the largest body of evidence, any significant association with *H pylori* was minimal after adjustments for confounding.^31^ In our study, the apparent protective effect of *H pylori* after adjustments for confounding was not surprising because *H pylori* will have been eradicated when found.

NSAID use might also have been under-reported, as NSAIDs can be bought over the counter from a pharmacy without a prescription, potentially explaining the low association between NSAIDs and bleeding in our study compared with a previous meta-analysis.^20^ However, we had higher recorded NSAID use than was reported in a recent national audit,^32^ and the studies used in the meta-analysis excluded patients with other known GIB risk factors.^20^ When we made the same exclusions in our study (Supplementary Table 2), or restricted to peptic ulcers, the association of bleeding with NSAIDs increased and became comparable with figures in the literature. With regard to over-the-counter use, nondifferential under-reporting has been shown to reduce the measured effect of prescribed medications.^33^ In our study, this would cause an underestimate of the effect of NSAIDs. However, in England, certain groups receive free prescriptions, such as patients older than 65 years or those with certain chronic diseases, and these groups have been shown to purchase far fewer medications over the counter than those who have to pay for prescriptions.^34,35^ When we restricted our analysis to those older than 65 years, thereby reducing confounding by over-the-counter medications, we found only a small reduction in the estimated PAF for comorbidity, but no change in PAF for NSAIDs. The final area of under-reporting that could affect our study was missing data for alcohol and smoking status, but these variables were not strong confounders of the association between comorbidity and bleeding and there was only a minimal effect on the PAF of comorbidity when missing data were imputed conditional on all available data and socioeconomic status.

We therefore believe that potential under-reporting of exposures does not explain the association we found between upper GIB and a general measure of comorbidity. This suggests that comorbidity itself, or other factors not included in our study that are associated with comorbidity, might be causing the association. It is possible that other medications not included in the study were responsible for some of this association, however, we are not aware of any additional prescribed or nonprescribed medication that would fulfill the requirements of common usage and a strong association with bleeding. Historically, nongastrointestinal comorbidity itself was commonly recognized as a risk factor for upper GIB.^7^ However, the concept of “stress ulceration” is no longer accepted, aside from patients on ITU who are exposed to severe acute physiological stresses from ventilation, coagulopathy, liver failure, renal failure, septic shock, or nutritional support.^9^ The physiological effects from chronic comorbidities in our study are unlikely to be as severe as those that occur on ITU and, therefore, what we are describing is likely to have a different mechanism than that seen in the ITU setting. Many potential mechanisms for our observed association can be hypothesized; for example, reduced epithelial microperfusion in cardiac failure,^36^ decreased oxygen levels in chronic obstructive pulmonary disease,^37,38^ poor nutritional status in many diseases, or the platelet and clotting dysfunction in end-stage renal failure.^27,39^ However, it is unlikely that there is a single mechanism that accounts for the association we found, but rather that multiple illnesses and mechanisms have a cumulative effect. This was shown by the graded effect of the Charlson Index and by Table 6, in which no individual disease accounted for the magnitude of the overall association with comorbidity.

Our findings contrast with current beliefs that the main burden of bleeding in the general population comes from known iatrogenic causes, such as NSAIDs prescribed for analgesia or antiplatelet agents prescribed for cardiac and cerebrovascular disease,^40^ and that this burden would be reduced by increasing PPI use.^41^ Instead, we have demonstrated that the extra contribution of these medications to bleeding cases was not large after considering the contributions of other risk factors present in the population. Therefore, simply increasing PPI prescriptions in patients on high-risk medications might not have as large an impact as previously thought.

In conclusion, the largest measurable burden of upper gastrointestinal hemorrhage in this study was attributed to nongastrointestinal comorbidity. In a proportion of patients, a bleed is an indicator of the burden of their comorbidity, and recognizing this will help guide management, particularly in the absence of modifiable gastrointestinal risk factors. However, our finding also explains why the incidence of nonvariceal bleeding is likely to remain high in an aging population, thereby necessitating continued acute gastroenterology service provision.

## Figures and Tables

**Supplementary Table 1:** Proportion of Cases Exposed 2 Months Before Bleed Date or Match Date Stratified by Coded Peptic Ulcer

	Peptic ulcer coded, *n*	Exposed, *%*	No peptic ulcer coded, *n*	Exposed, *%*
Charlson index^+^				
No comorbidity	883	18.3	2557	22.2
Single comorbidity	916	19.0	2306	20.0
Multiple or severe	3024	62.7	6669	57.8
Gastrointestinal				
Cirrhosis—none coded	4753	98.5	11,251	97.6
Cirrhosis only	17	0.4	46	0.4
Cirrhosis—varices	8	0.2	57	0.5
Cirrhosis—ascites	32	0.7	140	1.2
Cirrhosis—encephalopathy	13	0.3	38	0.3
Gastritis, duodenitis, or esophagitis	710	14.7	2341	20.3
Peptic ulcer	864	17.9	988	8.6
*Helicobacter pylori*	162	3.4	447	3.9
Angiodysplasia	1	0.0	5	0.0
Mallory-Weiss syndrome	11	0.2	85	0.7
Crohn's disease	19	0.4	95	0.8
GI cancer	254	5.3	920	8.0
Lifestyle				
Alcohol—not coded	3299	68.4	7727	67.0
Alcohol—non-drinker	97	2.0	278	2.4
Alcohol—ex-drinker	20	0.4	44	0.4
Alcohol—mentioned	284	5.9	693	6.0
Alcohol—over limits	1105	22.9	2658	23.0
Alcohol—complications	18	0.4	132	1.1
Smoking—not coded	2753	57.1	6434	55.8
Smoking—nonsmoker	646	13.4	1686	14.6
Smoking—ex-smoker	288	6.0	600	5.2
Smoking—passive	405	8.4	1050	9.1
Smoking—current	731	15.2	1762	15.3
Medications				
Aspirin	1831	38.0	3561	30.9
NSAIDs	1431	29.7	2467	21.4
COX II inhibitors	222	4.6	383	3.3
Clopidogrel	198	4.1	470	4.1
Oral steroids	428	8.9	1150	10.0
Anticoagulants	427	8.9	1190	10.3
SSRIs	460	9.5	1565	13.6
Other diagnoses				
Aortic stenosis	125	2.6	225	2.0
Repair of AAA	36	0.7	79	0.7
Dialysis	30	0.6	58	0.5
Confounders				
Previous upper GI procedure	817	16.9	2621	22.7
Previous PPI use	906	18.8	3679	31.9
Age (median and interquartile range)	75	64–83	72	54–82

AAA, Abdominal aortic aneurysm; SSRI, selective serotonin reuptake inhibitors. ^*a*^Nongastrointestinal comorbidity is catogorized as: Charlon Index = 0 is no comorbidity, Charlson Index = 1 single or comorbidity, and Charlson Index = 2 is multiple or severe comorbidity.

**Supplementary Table 2:** The Association of Medications With Upper Gastrointestinal Bleeding After Excluding Patients With Nonmedication Risk Factors (Age, Year, Practice, and Sex Matched)

	Crude OR	Adjusted^a^ OR	Lower 95% CI	Upper 95% CI
Aspirin	2.39	1.73	1.60	1.87
NSAIDs	2.80	1.78	1.64	1.93
COX II inhibitors	2.59	1.50	1.23	1.83
Clopidogrel	7.30	2.15	1.70	2.73
Oral steroids	1.23	1.23	1.08	1.41
Anticoagulants	4.83	2.26	1.99	2.57
SSRIs	2.78	1.52	1.34	1.71

SSRI, selective serotonin reuptake inhibitors.

a Adjusted for all other variables in Table and in Figure 1.

**Supplementary Table 3:** Sequential Population Attributable Fractions for Nonvariceal Upper Gastrointestinal Hemorrhage (NSAIDS Prescribed Between 1 Month and 1 Year Before Bleed)

	Sequential population attributable fractions,^a^^,^^b^*%*
Nongastrointestinal comorbidity	19.92
Gastrointestinal	
Cirrhosis	0.48
Gastritis, duodenitis, or esophagitis	1.86
Peptic ulcer	2.01
*Helicobacter pylori*	−0.06
Angiodysplasia	0.01
Mallory-Weiss syndrome	0.28
Crohn's disease	0.14
GI cancer	1.09
Lifestyle	
Alcohol use	2.83
Smoking	0.93
Medications	
Aspirin	2.91
NSAIDs	3.18
COX II inhibitors	0.36
Clopidogrel	0.32
Oral steroids	0.58
Anticoagulants	1.11
SSRIs	1.60
Other diagnoses	
Aortic stenosis	0.16
Repair of aorta	0.02
Dialysis	0.06

SSRI, selective serotonin reuptake inhibitors.

a Age, year, practice, and sex matched and adjusted for PPI use, previous upper gastrointestinal procedures, and age.

b The estimate in each row are calculated separately conditional on all the other variables in the model. They should therefore not be interpreted as summing over the column to 100%. Sequential PAF estimates the additional proportion of nonvariceal bleeding cases attributable to each risk factor after cases attributable to all the other risk factors in the model have been removed.

**Table 1:** Proportion of Cases and Controls Exposed 2 Months Before Bleed Date or Match Date

	Controls, *n*	Exposed, %	Cases, *n*	Exposed, %
Charlson Index^a^				
No comorbidity	30,194	37.0	3440	21.0
Single comorbidity	18,714	22.9	3222	19.7
Multiple or severe	32,728	40.1	9693	59.3
Gastrointestinal				
Cirrhosis—none coded	81,385	99.7	16,004	97.9
Cirrhosis only	65	0.1	63	0.4
Cirrhosis—varices	62	0.1	65	0.4
Cirrhosis—ascites	86	0.1	172	1.1
Cirrhosis—encephalopathy	38	0.0	51	0.3
Gastritis, duodenitis, or esophagitis	7904	9.7	3051	18.7
Peptic ulcer	3830	4.7	1852	11.3
*Helicobacter pylori*	1964	2.4	609	3.7
Angiodysplasia	14	0.0	6	0.0
Mallory-Weiss syndrome	34	0.0	96	0.6
Crohn's disease	222	0.3	114	0.7
GI cancer	2494	3.1	1174	7.2
Lifestyle				
Alcohol—not coded	61,536	75.4	11,026	67.4
Alcohol—nondrinker	1485	1.8	375	2.3
Alcohol—ex-drinker	176	0.2	64	0.4
Alcohol—mentioned	4317	5.3	977	6.0
Alcohol—over limits	14,073	17.2	3763	23.0
Alcohol—complications	49	0.1	150	0.9
Smoking—not coded	51,751	63.4	9187	56.2
Smoking—nonsmoker	11,666	14.3	2332	14.3
Smoking—ex-smoker	4075	5.0	888	5.4
Smoking—passive	5574	6.8	1455	8.9
Smoking—current	8570	10.5	2493	15.2
Medications				
Aspirin	18,079	22.1	5392	33.0
NSAIDs	12,722	15.6	3820	23.4
COX II inhibitors	1687	2.1	605	3.7
Clopidogrel	1297	1.6	668	4.1
Oral steroids	4135	5.1	1578	9.6
Anticoagulants	3799	4.7	1617	9.9
SSRIs	4813	5.9	2025	12.4
Other diagnoses				
Aortic stenosis	782	1.0	350	2.1
Repair of AAA	307	0.4	115	0.7
Dialysis	70	0.1	88	0.5
Confounders				
Previous upper GI procedure	10,471	12.8	3438	21.0
PPI	10,909	13.4	4585	28.0
Age (median and interquartile range)	73	57–82	72	57–81

AAA, Abdominal aortic aneursym; SSRI, selective serotonin reuptake inhibitors.

a Nongastrointestinal comorbidity is catogorized as: Charslon Index = 0 is no comorbidity, Charlson Index = 1 single or comorbidity, and Charlson Index = 2 is multiple or severe comorbidity.

**Table 2:** Adjusted Model for Nonvariceal Upper Gastrointestinal Bleeding All Cases (Age, Year, Practice, and Sex Matched)

	Adjusted OR	Lower 95% CI	Upper 95% CI
Charlson Index			
No comorbidity	1.00	1.00	1.00
Single comorbidity	1.43	1.35	1.52
Multiple or severe	2.26	2.14	2.38
Gastrointestinal			
Cirrhosis—none	1.00	1.00	1.00
Cirrhosis only	3.89	2.61	5.77
Cirrhosis—varices	3.75	2.51	5.61
Cirrhosis—ascites	5.96	4.46	7.96
Cirrhosis—encephalopathy	5.05	3.14	8.10
Gastritis, duodenitis, or esophagitis	1.46	1.39	1.55
Peptic ulcer	2.11	1.98	2.26
*Helicobacter pylori*	0.96	0.86	1.07
Angiodysplasia	1.67	0.58	4.80
Mallory-Weiss syndrome	12.39	8.16	18.82
Crohn's disease	2.19	1.71	2.81
GI cancer	2.13	1.97	2.31
Lifestyle			
Alcohol—not	1.00	1.00	1.00
Alcohol—nondrinker	1.25	1.10	1.42
Alcohol—ex-drinker	1.39	1.01	1.92
Alcohol—mentioned	1.05	0.96	1.14
Alcohol—over limits	1.42	1.35	1.49
Alcohol—complications	9.33	6.48	13.44
Smoking—not	1.00	1.00	1.00
Smoking—non-smoker	0.97	0.92	1.04
Smoking—ex-smoker	0.94	0.86	1.02
Smoking—passive	1.03	0.95	1.11
Smoking—current	1.29	1.22	1.37
Medications			
Aspirin	1.50	1.43	1.57
NSAIDs	1.59	1.52	1.66
COX II inhibitors	1.52	1.37	1.69
Clopidogrel	1.74	1.57	1.94
Oral steroids	1.38	1.29	1.48
Anticoagulants	1.94	1.81	2.08
SSRIs	1.72	1.62	1.83
Other diagnoses			
Aortic stenosis	1.58	1.38	1.82
Repair of AAA	1.29	1.02	1.64
Dialysis	3.59	2.55	5.05
Confounders			
Previous upper GI procedure	1.10	1.04	1.15
PPI	1.59	1.52	1.67
Age	1.09	1.08	1.10

AAA, Abdominal aortic aneurysm; SSRI, selective serotonin reuptake inhibitors.

**Table 3:** Adjusted Model for Nonvariceal Upper Gastrointestinal Bleeding Stratified by Coding of Peptic Ulcer (Age, Year, Practice, and Sex Matched)

	Peptic ulcer			Nonpeptic ulcer		
	Adjusted OR	Lower 95% CI	Upper 95% CI	Adjusted OR	Lower 95% CI	Upper 95% CI
Charlson Index^a^						
No comorbidity	1.00	1.00	1.00	1.00	1.00	1.00
Single comorbidity	1.45	1.30	1.62	1.42	1.33	1.52
Multiple or severe	2.28	2.06	2.52	2.27	2.13	2.42
Gastrointestinal						
Cirrhosis—none	1.00	1.00	1.00	1.00	1.00	1.00
Cirrhosis only	3.98	2.03	7.80	3.80	2.30	6.27
Cirrhosis—varices	2.33	0.92	5.94	4.15	2.63	6.54
Cirrhosis—ascites	4.67	2.63	8.29	6.85	4.85	9.65
Cirrhosis—encephalopathy	3.16	1.39	7.20	6.66	3.70	12.01
Gastritis, duodenitis, or esophagitis	1.22	1.10	1.36	1.58	1.48	1.68
Peptic ulcer	4.36	3.92	4.85	1.37	1.25	1.49
*Helicobacter pylori*	1.04	0.85	1.27	0.94	0.83	1.06
Angiodysplasia	1.71	0.16	18.64	1.49	0.44	5.00
Mallory Weiss syndrome	3.75	1.43	9.84	16.54	10.23	26.77
Crohns disease	1.18	0.68	2.05	2.65	1.99	3.54
GI cancer	1.45	1.23	1.69	2.45	2.23	2.70
Lifestyle						
Alcohol—not	1.00	1.00	1.00	1.00	1.00	1.00
Alcohol—nondrinker	1.14	0.89	1.47	1.30	1.11	1.51
Alcohol—ex-drinker	1.58	0.89	2.81	1.30	0.88	1.93
Alcohol—mentioned	1.02	0.87	1.20	1.04	0.94	1.16
Alcohol—over limits	1.34	1.22	1.47	1.45	1.36	1.54
Alcohol—complications	3.88	1.70	8.87	11.85	7.76	18.10
Smoking—not	1.00	1.00	1.00	1.00	1.00	1.00
Smoking—nonsmoker	0.99	0.88	1.11	0.96	0.90	1.04
Smoking—ex-smoker	0.96	0.82	1.13	0.92	0.83	1.03
Smoking—passive	0.95	0.82	1.09	1.06	0.97	1.16
Smoking—current	1.35	1.21	1.51	1.28	1.19	1.37
Medications						
Aspirin	1.69	1.56	1.82	1.42	1.34	1.50
NSAIDs	2.21	2.04	2.39	1.37	1.29	1.45
COX II inhibitors	1.81	1.51	2.17	1.42	1.24	1.62
Clopidogrel	2.04	1.68	2.48	1.70	1.49	1.93
Oral steroids	1.31	1.16	1.49	1.40	1.29	1.51
Anticoagulants	1.67	1.47	1.90	2.10	1.94	2.28
SSRIs	1.47	1.30	1.66	1.84	1.71	1.97
Other diagnoses						
Aortic stenosis	1.79	1.41	2.26	1.46	1.23	1.75
Repair of AAA	1.33	0.87	2.04	1.27	0.95	1.68
Dialysis	5.56	2.95	10.48	2.92	1.94	4.41
Confounders						
Previous upper GI procedure	0.88	0.80	0.98	1.20	1.13	1.28
PPI	0.82	0.74	0.91	2.01	1.90	2.13
Age	1.10	1.08	1.11	1.09	1.08	1.10

AAA, Abdominal aortic aneursym; SSRI, selective serotonin reuptake inhibitors.

a Nongastrointestinal comorbidity is catogorized as: Charlon Index = 0 is no comorbidity, Charlson Index = 1 single or comorbidity, and Charlson Index = 2 is multiple or severe comorbidity.

**Table 4:** Sequential Population Attributable Fractions for Nonvariceal Upper Gastrointestinal Hemorrhage (All Cases)

	Sequential population attributable fractions^a^^,^^b^	
	*%*	95% CI
Nongastrointestinal comorbidity	19.80	18.43 to 21.18
Gastrointestinal		
Cirrhosis	0.49	0.41 to 0.57
Gastritis, duodenitis or esophagitis	1.98	1.66 to 2.30
Peptic ulcer	2.05	1.81 to 2.28
*Helicobacter pylori*	−0.04	−0.15 to 0.08
Angiodysplasia	0.01	−0.01 to 0.02
Mallory-Weiss syndrome	0.29	0.22 to 0.37
Crohn's disease	0.14	0.08 to 0.19
GI cancer	1.11	0.96 to 1.27
Lifestyle		
Alcohol use	2.89	2.39 to 3.39
Smoking	0.83	0.27 to 3.42
Medications		
Aspirin	2.95	2.54 to 3.36
NSAIDs	3.07	2.72 to 3.42
COX II inhibitors	0.33	0.23 to 0.44
Clopidogrel	0.34	0.26 to 0.43
Oral steroids	0.59	0.44 to 0.74
Anticoagulants	1.19	1.04 to 1.35
SSRIs	1.58	1.36 to 1.80
Other diagnoses		
Aortic stenosis	0.16	0.10 to 0.22
Repair of aorta	0.03	0.00 to 0.06
Dialysis	0.07	0.04 to 0.09

SSRI, selective serotonin reuptake inhibitors.

a Age, year, practice, and sex matched and adjusted for PPI use, previous upper gastrointestinal procedures, and age.

b The estimate in each row are calculated separately conditional on all the other variables in the model. They should therefore not be interpreted as summing over the column to 100%. Sequential PAF estimates the additional proportion of nonvariceal bleeding cases attributable to each risk factor after cases attributable to all the other risk factors in the model have been removed.

**Table 5:** Sequential Population Attributable Fractions for Nonvariceal Upper Gastrointestinal Hemorrhage Strafied by Coding for Peptic Ulcer

	Sequential population attributable fractions,^a^^,^^b^*%*	
	Peptic ulcer	Nonpeptic ulcer
Nongastrointestinal comorbidity	18.44	20.50
Gastrointestinal		
Cirrhosis	0.32	0.57
Gastritis, duodenitis, or esophagitis	0.69	2.74
Peptic ulcer	5.31	0.69
*Helicobacter pylori*	0.05	−0.07
Angiodysplasia	0.01	0.00
Mallory-Weiss syndrome	0.06	0.39
Crohn's disease	0.02	0.19
GI cancer	0.35	1.48
Lifestyle		
Alcohol use	1.93	3.30
Smoking	0.80	0.81
Medications		
Aspirin	3.99	2.42
NSAIDs	5.40	2.00
COX II inhibitors	0.47	0.28
Clopidogrel	0.38	0.35
Oral steroids	0.36	0.66
Anticoagulants	0.78	1.41
SSRIs	0.74	2.02
Other diagnoses		
Aortic stenosis	0.22	0.12
Repair of aorta	0.02	0.03
Dialysis	0.09	0.05

SSRI, selective serotonin reuptake inhibitors.

a Age, year, practice, and sex matched and adjusted for PPI use, previous upper gastrointestinal procedures and age.

b The estimate in each row are calculated separately conditional on all the other variables in the model. They should therefore not be interpreted as summing over the column to 100%. Sequential PAF estimates the additional proportion of nonvariceal bleeding cases attributable to each risk factor after cases attributable to all the other risk factors in the model have been removed.

**Table 6:** The Adjusted^a^ Association of the Component Comorbidities of Charlson Index With Nonvariceal Bleeding

	Proportion of cases exposed, *%*	OR^a^	Lower 95% CI	Upper 95% CI	PAF,^b^*%*
Myocardial infarction	13.98	1.04	0.97	1.10	0.12
Congestive cardiac disease	19.90	1.49	1.41	1.58	1.95
Peripheral vascular disease	11.17	1.31	1.23	1.41	0.70
Dementia	8.98	1.40	1.30	1.50	1.00
Chronic pulmonary disease	31.80	1.11	1.06	1.16	1.10
Cerebrovascular disease	23.1	1.13	1.08	1.19	0.80
Rheumatological disease	10.13	1.06	0.99	1.13	0.17
Uncomplicated diabetes	17.88	1.01	0.96	1.06	0.04
Hemiplegia	4.73	1.79	1.62	1.97	0.67
Renal disease	14.42	1.71	1.61	1.82	1.74
Diabetes with complications	12.30	1.00	0.94	1.06	−0.01
Any malignancy	13.11	1.21	1.14	1.28	0.78
Lymphoproliferative disorders	2.21	1.95	1.70	2.24	0.43
Metastatic solid tumour	5.89	2.35	2.14	2.57	1.29
HIV/AIDS	0.06	0.69	0.31	1.55	−0.00

HIV/AIDS, human immunodeficiency virus/acquired immunodeficiency syndrome.

a Adjusted for all other variables in this Table and Table 2 and matched for year, practice, and sex.

b Sequential population attributable fractions: The estimates in each row are calculated separately conditional on all the other variables in the model. They should therefore not be interpreted as summing over the column to 100%. Sequential PAF estimates the additional proportion of nonvariceal bleeding cases attributable to each risk factor after cases attributable to all the other risk factors in the model have been removed.
